# Estimating minimal clinically important differences in primary elective total elbow arthroplasty using distribution-based methods

**DOI:** 10.1016/j.jsea.2026.100057

**Published:** 2026-06-25

**Authors:** Dimitris Challoumas, Tin-Ning Wong, Neal Millar, Christopher A. Peach

**Affiliations:** a Wrightington Upper Limb Unit, Wrightington, Wigan & Leigh NHS Foundation Trust, Wigan, UK; b School of Infection and Immunity, University of Glasgow, Glasgow, UK; c Trauma & Orthopaedics, Manchester University Foundation Trust, Manchester, UK

**Keywords:** MCID, PROM, Clinical significance, Elbow replacement, Function, ROM

## Abstract

**Background:**

Minimal clinically important differences (MCIDs) following primary total elbow arthroplasty (TEA) remain undefined, limiting interpretation of clinical benefit. This study aimed to determine TEA-specific distribution-based MCIDs from baseline variability in primary elective TEA cohorts and identify previously published MCIDs across elbow conditions.

**Methods:**

A systematic search identified primary elective TEA studies reporting baseline variability statistics. Pooled standard deviations (SD) estimated MCIDs via the half-SD method (all measures) and the one standard error of measurement method (multi-item scales). A scoping review identified published anchor- or distribution-based MCIDs. Outcomes assessed: pain visual analog scale, Mayo Elbow Performance Score (MEPS), Disabilities of Arm, Shoulder and Hand, Quick Disabilities of Arm, Shoulder and Hand, Oxford Elbow Score, and elbow range of motion.

**Results:**

Fifty-six studies contributed to baseline data. Half-SD-derived MCIDs were 1.0 point for pain visual analog scale, 11 points for MEPS, 15° for flexion-extension arc, and 20° for pronation-supination arc; the one-standard error of measurement-derived MCID for MEPS was 16 points. The scoping review identified no TEA-specific MCID studies and 9 across other elbow conditions, with wide variability.

**Conclusion:**

These TEA-specific, distribution-based MCID estimates for pain, function, and range of motion offer a pragmatic framework for outcome interpretation, shared decision-making, and future research.

Total elbow arthroplasty (TEA) is an established treatment option for patients with end-stage elbow pathology, including inflammatory arthritis (IA), complex fractures, post-traumatic arthritis, osteoarthritis, and salvage following failed fixation. While the number of TEAs performed acutely for unreconstructible fractures is increasing, that of elective TEAs is steadily decreasing, predominantly due to the advances in the medical management of rheumatoid arthritis (RA).^27^ Despite its efficacy in improving functional outcomes, complication and revision rates remain higher than other large-joint arthroplasties, making careful evaluation of clinical benefit essential.^27^ Patient-reported outcome measures (PROMs) are therefore central to assessing the effectiveness of TEA and informing shared decision-making, audit, and research.^3^^,^^8^ While historically PROM reporting in TEA has been inconsistent, recent systematic reviews demonstrate increasing use of validated measures such as the Mayo Elbow Performance Score (MEPS), the Disabilities of the Arm, Shoulder and Hand (DASH) and QuickDASH scales, and the Oxford Elbow Score (OES) across large multicenter cohorts.^32^

While statistically significant improvements in PROMs may be demonstrated following TEA, statistical significance alone does not equate to clinical relevance. The concept of the minimal clinically important difference (MCID) was developed to address this gap, representing the smallest change in an outcome measure perceived as beneficial by patients and sufficient to justify a change in management.^32^ Determining accurate MCID values is critical for interpreting outcomes, comparing interventions, and designing adequately powered clinical studies.^20^ Despite this importance, MCID thresholds specific to TEA populations remain under-reported, limiting the interpretability of meaningful change after elbow arthroplasty.

MCID can be estimated using anchor- or distribution-based methods. Anchor-based approaches link changes in PROM scores to an external criterion, such as patient satisfaction or global rating of change (GROC), and are often considered conceptually intuitive.^6^ However, they require reliable anchors, are susceptible to recall bias, and may be limited by sample size and anchor validity. In contrast, distribution-based methods use statistical characteristics of outcome scores to estimate clinically meaningful change, offering a pragmatic alternative when suitable anchors are unavailable.^39^ These approaches are particularly relevant in retrospective datasets and registry-based studies, where anchor data are frequently lacking.

Among distribution-based methods, the use of one standard error of measurement (one SEM) and half a standard deviation (half SD) are widely accepted and commonly applied. The SEM reflects the measurement precision of an instrument and estimates the smallest change beyond measurement error, thereby providing a conservative threshold for clinically meaningful improvement.^39^ The half SD method is based on empirical observations that half a SD corresponds to a moderate effect size and is often perceived by patients as a meaningful change across a range of health-related quality-of-life measures.^29^ Both approaches have been validated across multiple musculoskeletal conditions and are recommended when anchor-based methods are impractical or when complementary estimates are required.^6^

In the context of TEA, the heterogeneity of indications, relatively low procedural volumes, and limited PROM datasets present challenges to MCID determination. Recent midterm cohort studies demonstrate meaningful improvements across function and quality of life instruments following contemporary TEA designs, underscoring the need for condition-specific MCID thresholds for these measures.^14^ Additionally, emerging work on simplified PROM metrics such as the Single Assessment Numeric Evaluation in elbow arthroplasty suggests alternative approaches to capture patient perspectives that may eventually inform MCID work.^37^ There remains a need for robust, data-driven MCID estimates derived specifically from TEA populations using validated outcome measures.

The aim of this study is dual: a) to identify and present previously published MCIDs of commonly used TEA outcome measures deriving from either distribution- or anchor-based methods; b) to determine MCID values for the same outcome measures following TEA using distribution-based methods, specifically one SEM and half SD. By providing TEA-specific, statistically grounded MCID thresholds, this work seeks to improve interpretation of clinical outcomes, support future research design, and enhance the meaningful evaluation of patient benefit following elbow arthroplasty.

## Materials and methods

This study consisted of 2 distinct parts. The first part used baseline variability statistics from studies on primary elective TEA to determine distribution-based MCIDs for commonly used outcome measures. The second part was a scoping review aimed at identifying previously published MCIDs in TEA or other elbow conditions. Ethical Committee or institutional review board approval was not required as no human participants, data or tissue are involved in this study.

### Eligibility

For the first part, studies of any methodological type (comparative or noncomparative) were eligible if they reported baseline variability statistics of outcome measures of interest in patients undergoing primary elective TEA. Any type of variability statistic is eligible: SD, confidence interval (CI), interquartile range (IQR), range, and standard error of mean. Studies on TEA for trauma were excluded as baseline pre-operative data were not possible, and so were those on revision TEA and elbow hemiarthroplasty, as we wanted to determine MCIDs for primary elective TEA only.

For the second part (scoping review), we were interested in studies of any type reporting MCID values on outcome measures commonly used in elbow conditions that were derived from either distribution- or anchor-based method. The primary goal was to identify studies that calculated MCIDs in TEA so that we could directly compare with our estimated MCID values from the first part of our study; however, other elbow conditions were also sought.

### Study identification

For the first part, a comprehensive search was performed in PubMed, Medline, and EMBASE (via Ovid) in April 2025 using keywords: “elbow arthroplasty” and “elbow replacement.” OpenGrey was also searched for unpublished studies. Reference and citation list of included studies were reviewed for further eligible publications. A total of 3,378 studies were identified after removing duplicates. Titles, abstracts, and full texts were screened by 2 independent reviewers. Although no date filters were used initially, we subsequently decided to focus on studies published from 2000 onward; therefore, earlier studies were excluded. We were not interested in more historical data published before 2000, as PROMs had not been developed yet, and the patient characteristics and variability statistics of those undergoing primary elective TEA then may not reflect contemporary practice.

For the second part (scoping review), the following search terms and their combinations were used without date criteria or any other restrictions: “MCID,” “MID,” “MIC,” “Minim∗ clinically important difference,” “Minim∗ important difference,” “Minim∗ important change,” “elbow arthroplasty,” “elbow replacement,” “elbow,” “MEPS,” “Mayo Elbow Performance Score,” “Mayo Elbow Performance Index,” “DASH,” “Disabilities of arm, shoulder, hand,” “Q-DASH,” “Quick-DASH,” “OES,” “Oxford Elbow Score,” “pain,” “visual analogue scale,” “range of movement,” “elbow flexion,” “elbow extension,” “forearm pronation,” “forearm supination”, “forearm rotation.”

### Outcome measures

Patient-reported pain visual analog scale (VAS, 0-10), total arc of range of motion (ROM) for flexion-extension (FE) and pronation-supination (PS), and the following functional outcomes were used for determination of MCIDs: MEPS, the DASH scale, the QuickDASH scale, and the OES.

### Data handling

For the first part, population size and baseline mean and SD (or other variability statistics) data for each outcome measure of interest were extracted from each eligible study and tabulated into Microsoft Word. Follow-up post-operative study data were ignored as they were irrelevant for the purposes of our study. MCIDs were estimated for the overall population but also for different populations based on indication for TEA (RA/IA vs. osteoarthritis vs. post-traumatic arthritis) where sufficient data were available for the outcome measure of interest (at least 2 studies providing data on baseline variability statistics).

The MCIDs of each outcome measure of interest were calculated as follows:

a) For all outcome measures (single-item and multi-item), the MCID was estimated to be equal to half the pooled SD at baseline based on the rule of half SD; where the reliability of the instrument is very high (which is generally the case for single-item outcome measures, such as pain VAS), using the one SEM rule would result in a very small value of MCID, and therefore the half SD threshold is a more stringent criterion.^29^
b) For multi-item PROMs (e.g., DASH), the one SEM rule was also used to calculate the MCID to see how that compares to the corresponding value deriving from the rule of half SD.^29^ Cronbach's alpha was used as an internal consistency measure in the equation. This approach yields a more conservative value, which represents the smallest change in the instrument that is not due to measurement error related to reliability.

The SEM is a statistic used to estimate how reliably an instrument measures an individual's “true score.” The Cronbach's alpha, which measures the internal consistency of an instrument, is the degree of the inter-relatedness among the items of the instrument.^26^ In contrast to test-retest statistics (eg, intraclass correlation coefficient), using Cronbach's alpha is especially useful when the assessed parameters can change over short periods of time.^31^

We standardized pain VAS scores by using a 10-point scale (0-10) as this was the most used scale; where 0-100 scale was used by studies, reported values were converted to their equivalent on a 0-10 scale by dividing them by “10.”

For the second part (scoping review), relevant MCIDs of interest were simply extracted from the identified papers and tabulated in Microsoft Word (Microsoft Corporation, 2021) for presentation purposes.

### Statistical analysis

All variability statistics were converted to SDs. When IQRs or data ranges were reported, the SD was calculated as the IQR divided by 1.35 and the length of the range divided by 4, respectively. When CIs of means were reported, SDs were calculated by dividing the length of the CI by 3.92 and then multiplying the square root of the sample size. When standard error of mean was given, this was converted to SD by multiplying it by the square root of the sample size.

Pooled SDs were calculated with the following formula: SD_pooled_ = √(SD_1_^2^[*n*_1_-1]) + (SD_2_^2^[*n*_2_-1]) + … + (SD_*k*_^2^[*n*_*k*_-1])/(*n*_1_ + *n*_2_ + … + *n*_*k*_ – *k*), where *n* indicates sample size and *k*, the number of samples.

SEM for each multi-item PROM was calculated with the formula: SEM = SD√ (1-r_xx_), where r_xx_ is the internal consistency of the scale (Cronbach's alpha coefficient) and SD the pooled SD for that instrument at baseline from the included RCTs. The Cronbach's alpha coefficient for each instrument was obtained from reliability data in the published literature and where more than one studies reported internal consistency values (eg different language versions of the instrument), the average of these values was calculated and used. For all functional outcomes, priority was given to the British version of the instrument. All computed MCIDs were rounded up to the next higher integer (eg a value of 10.2 was rounded up to 11).

## Results

For the first part of the study, from an initial total of 75 TEA studies initially identified from the literature search, 56 studies (n = 2,563 TEAs, mean age 59 years, mean female proportion 76%) contributed data to baseline pooled SD calculation for any outcome measure and were included. Thirty-six studies included patients with RA/IA only, 2 with post-traumatic arthritis only, one with primary osteoarthritis only, and the remaining 17 included patients with mixed indications. The most used functional scales in the identified studies were the MEPS, the DASH, the QuickDASH, and the OES.

Cronbach's alpha values for the multi-item instruments were pre-set as follows: MEPS 0.43, DASH 0.97, QuickDASH 0.93, and OES 0.88.^1^^,^^9^^,^^10^^,^^13^^,^^16^, ^17^, ^18^^,^^24^^,^^25^^,^^33^

For the scoping review part, a total of 9 studies^10^^,^^11^^,^^19^^,^^21^^,^^28^^,^^34^^,^^36^^,^^38^^,^^40^ that reported MCIDs of outcomes of interest were used. The most used distribution-based method was the half SD method—some of them used the baseline SD and some others the SD of the improvement from baseline to follow-up. Where studies used an anchor-based method that was predominantly linked to a GROC scale—the mean change in score of those that reported “no change” or “somewhat dissatisfied” was subtracted from that of those who reported “somewhat satisfied.”

### Minimal clinically important difference determination

From the outcome measures of interest (pain VAS, MEPS, DASH, QuickDASH, OES, FE ROM, and PS ROM), the following had baseline variability statistics in at least 2 studies and could be used for MCID computation: pain VAS, MEPS, FE ROM, and PS ROM (all except for OES). DASH and QuickDASH only had one study each with 21 and 25 patients, respectively, contributing to baseline SD calculation; therefore; they were excluded.

Table I shows the number of studies and TEAs that contributed data for each outcome measure of interest for the overall population and the resulting baseline pooled SD, as well as the computed MCID for each outcome measure using the 2 distribution-based methods: one SEM and half SD. Table II shows the subgroup analyses results for RA/IA patients only. All estimated MCIDs were the same for the overall population and RA/IA patients specifically, other than FE arc, which was 15 and 14°, respectively. No other subgroup analyses could be performed for other populations, as available data were insufficient.

**Table I:** Summary and results of included studies.

Parameter	Pain VAS	MEPS	FE arc	PS arc
No of studies	8	29	36	24
Total no of TEA	207	953	1,316	862
Pooled SD	2.0	20.8	28.3	38.5
Cronbach's alpha used	-	0.43	-	-
Half SD method	1.0	11	15	20
One SEM method	-	16	-	-

*VAS*, visual analog scale; *MEPS*, Mayo Elbow Performance Score; *FE*, flexion-extension; *PS*, pronation-supination; *SD*, standard deviation; *SEM*, standard error of measurement; *TEA*, total elbow arthroplasty.

**Table II:** Summary and results of included studies that included patients with rheumatoid/inflammatory arthritis only.

Parameter	Pain VAS	MEPS	FE arc	PS arc
No of studies	3	18	23	16
Total no of TEA	92	637	993	622
Pooled SD	2.0	21.4	26.6	40.1
Cronbach's alpha used	-	0.43	-	-
Half SD method	1.0	11	14	20
One SEM method	-	16	-	-

*VAS*, visual analog scale; *MEPS*, Mayo Elbow Performance Score; *FE*, flexion-extension; *PS*, pronation-supination; *SD*, standard deviation; *SEM*, standard error of measurement; *TEA*, total elbow arthroplasty.

### Scoping review

Table III provides a summary of the MCIDs of commonly used elbow outcome measures determined by previously published studies. No studies were identified that computed MCIDs for TEA specifically for the outcome measures of interest.

**Table III:** Summary of the Minimal clinically important differences (MCIDs) of commonly used elbow outcome measures.

Studies	Condition	Method used	Pain (VAS)	DASH	QuickDASH	MEPS	OES	FE ROM	PS ROM
Challoumas et al (2023)^5^	Lateral elbow tendinopathy	Distribution-based (half baseline SD)	1.0	8.9					
		Distribution-based (one SEM)		4.1					
Farzad et al (2022)^11^	Lateral elbow tendinopathy	Anchor-based		18					
Ben et al (2025)^2^	Arthroscopic elbow arthrolysis	Distribution-based (half SD improvement)	1.2			9.5		15	
Iordens et al (2017)^19^	Simple elbow dislocation	Anchor-based			3.5		8.2		
Karjalainen et al (2023)^21^	Lateral elbow tendinopathy	Anchor-based			7-9	14-20			
Smith-Forbes et al (2016)^34^	Lateral elbow tendinopathy	Anchor-based			15.8				
Sun et al (2021)^36^	Open elbow arthrolysis	Anchor-based				12.2		25	
		Distribution-based (half SD improvement)				8.3		14.1	
van Es et al (2024)^10^	Corrective osteotomy for distal radius malunion	Distribution-based (half baseline SD)							19
Wänström et al (2024)^38^	Elbow trauma	Anchor-based			11.4		9.3		
		Distribution-based (one SEM)			11.4		12.1		

*VAS*, visual analog scale; *MEPS*, Mayo Elbow Performance Score; *FE*, flexion-extension; *PS*, pronation-supination; *SD*, standard deviation; *SEM*, standard error of measurement; *DASH*, Disabilities of Arm, Shoulder and Hand; *OES*, Oxford Elbow Score; *ROM*, range of motion.

### Pain

For patient-reported pain on the VAS scale (0-10 points), Challoumas et al (2023)^5^ suggested a MCID of 1.0 point for lateral elbow tendinopathy derived with a distribution-based method (half SD_baseline_). Ben et al (2025)^2^ also used a distribution-based method (half SD_improvement_) and determined the MCID for pain to be 1.2 points for arthroscopic arthrolysis of the elbow for stiffness.

#### Disabilities of Arm, Shoulder and Hand

Two studies attempted to determine DASH MCIDs in lateral elbow tendinopathy. Through an anchor-based method, Farzad et al (2022)^11^ computed a DASH MCID of 18 points. Substantially lower DASH MCIDs were suggested by Challoumas et al (2023)^5^ who used 2 distribution-based methods; when the half SD method was used, the estimated MCID was 8.9, while this was 4.1 when the one SEM rule was used.

#### Quick Disabilities of Arm, Shoulder and Hand

Four studies that used anchor-based methods reported estimated QDASH MCIDs for elbow conditions. Smith-Forbes et al (2016)^34^ estimated this to be 15.8 points with the use of GROC in lateral elbow tendinopathy, which was approximately double the MCID range of 7-9 points determined by Karjalainen et al (2023)^21^ who used a mixture of anchor-based techniques.

Wänström et al (2024)^38^ reported an estimated QuickDASH MCID of 11.4 points in patients recovering from elbow trauma (mostly radial head fractures). This value was determined using the GROC anchor-based method; however; the exact same value was computed as the smallest detectable change, which was an estimation of the SEM of the instrument.

Finally, Iordens et al (2017)^19^ used data from a RCT on simple elbow dislocation and using the GROC anchor-based method, estimated the QDASH MCID to be 3.5 points.

#### Oxford Elbow Score

The same 2 studies by Wänström et al (2024)^38^ and Iordens et al (2017)^19^ provided an estimation of OES MCID for elbow trauma and simple elbow dislocation, respectively. Their anchor-based method produced a MCID estimation of 9.3 points and 8.2, respectively, whereas when the one SEM method was also used by Wänström et al (2024),^38^ they determined the smallest detectable change to be 12.1 points.

#### Mayo Elbow Performance Score

Two studies attempted to estimate a MEPS MCID in elbow arthrolysis. Sun et al (2021)^36^ suggested values of 12.2 points (anchor-based method, GROC) and 8.3 points (distribution-based method, half SD_improvement_) as MCID after open arthrolysis. Ben et al (2025)^2^ reported an estimated MCID of 9.5 points after arthroscopic arthrolysis using a distribution-based method (half SD_improvement_). Finally, for lateral elbow tendinopathy, Karjalainen et al (2023)^21^ suggested a range of MCID of 14-20 using a mixture of anchor-based methods.

#### Flexion-extension range of motion

The same 2 studies on elbow arthrolysis provided insights into total arc of FE ROM MCID. The estimated value of 15° by Ben et al (2025)^2^ after arthroscopic arthrolysis deriving from their distribution-based method was very similar to the 14.1° reported by Sun et al (2021)^36^ after open elbow arthrolysis, using the same distribution-based method. When the latter study used an anchor-based technique, the estimated MCID was higher at 25 points.

### Pronation-supination range of motion

The study by van Es et al (2024)^10^ used a distribution-based method (half SD_baseline_) for their recommended MCID of 19° in total arc of PS ROM after corrective osteotomy for distal radius malunion. No other relevant published data were identified.

## Discussion

This study provides TEA-specific estimates of MCIDs for commonly used elbow outcome measures using robust, distribution-based methods, while also synthesizing previously published MCIDs across elbow conditions. The principal finding is that clinically meaningful change thresholds for pain, function, and ROM following primary elective TEA can be reasonably approximated using baseline variability statistics, despite the inherent challenges posed by low procedure volumes and heterogeneous indications. By grounding MCID estimates in contemporary elective TEA cohorts, this work addresses an important gap in outcome interpretation and provides clinically useful benchmarks for future research and audit.

A key strength of this study lies in its focus on primary elective TEA, excluding trauma and revision cohorts. This distinction is critical, as baseline disability, pain, and variability differ substantially between elective and acute fracture populations, where pre-operative PROM collection is often not feasible. Many previously published MCID estimates in elbow surgery derive from trauma-related or soft tissue conditions such as lateral elbow tendinopathy, arthrolysis, or elbow dislocation, limiting their applicability to arthroplasty populations.^2^^,^^5^^,^^10^^,^^11^^,^^19^^,^^21^^,^^34^^,^^36^^,^^38^ Our findings, therefore, complement the existing literature by providing TEA-relevant MCID thresholds that better reflect the expectations and baseline impairment of patients undergoing elective elbow replacement.

Using the half SD method, the estimated MCID for pain VAS was 1.0 point on a 0-10 scale. This aligns closely with previously reported values across elbow conditions, including lateral elbow tendinopathy and elbow arthrolysis, where MCIDs between 1.0 and 1.2 points have been suggested using distribution-based approaches.^33^ This consistency across disparate elbow pathologies supports the face validity of pain MCIDs and suggests that a 1-point reduction on the VAS represents a reasonable threshold for clinically meaningful pain improvement following TEA.

For functional outcome measures, the estimated MCIDs varied by instrument and method. For the MEPS, a half SD-based MCID of 11 points and a one SEM-based MCID of 16 points were identified. These values fall within the range reported in prior elbow arthrolysis and tendinopathy studies, where MCIDs between 8 and 14 points have been suggested depending on the method used.^7^^,^^22^ The relatively low Cronbach's alpha used for the MEPS (0.43) reflects its composite nature, incorporating pain, motion, stability, and function, and may partly explain the discrepancy between 2 distribution-based estimates. Importantly, this variability underscores the limitations of MEPS as an instrument and reinforces the need for cautious interpretation of small changes, particularly when used as a primary endpoint. Based on our data, MEPS is the most used functional scale in primary elective TEA studies.

For DASH and QuickDASH instruments, only single studies contributed data, and therefore, we did not formally estimate MCIDs. Based on the scoping review, anchor-based MCIDs for DASH and QuickDASH ranged from 8 to 18 points depending on condition and methodology.^12^^,^^15^^,^^33^^,^^35^

ROM outcomes demonstrated MCIDs of 15° for FE arc and 20° for PS arc using the half SD method. These estimates are consistent with prior work in elbow arthrolysis and corrective osteotomy, where MCIDs for FE arc ranged from 14° to 25° depending on the method used.^23^ The alignment of our findings with these studies suggests that baseline variability in elective TEA cohorts yields clinically intuitive thresholds for motion improvement, supporting their use in both clinical assessment and research reporting.

The scoping review highlights substantial heterogeneity in previously published MCID values, particularly those derived using anchor-based methods. Across DASH, QuickDASH, MEPS, and OES, anchor-based MCIDs were frequently higher than distribution-based estimates and varied widely depending on the anchor used, patient population, and definition of meaningful change. This observation is consistent with prior methodological work demonstrating that anchor-based MCIDs are sensitive to recall bias, anchor selection, and patient expectations.^6^^,^^30^

In the context of TEA, where anchor data such as GROC are rarely collected and sample sizes are often modest, distribution-based methods provide a pragmatic and reproducible alternative. While anchor-based approaches remain conceptually attractive, particularly for capturing patient perception, their limitations in retrospective and registry-based datasets make them difficult to apply reliably. Our findings, therefore, support the use of distribution-based MCIDs, particularly the half SD method, as a reasonable and transparent standard for interpreting PROM changes following TEA.

The MCID thresholds proposed in this study have several important implications. Clinically, they provide surgeons with tangible benchmarks for interpreting post-operative improvement and counseling patients regarding expected benefit. For example, a post-operative DASH improvement of 10 points would exceed the half SD-derived MCID and could reasonably be considered clinically meaningful, even if anchor-based thresholds from other elbow conditions suggest higher values. Similarly, improvements in FE arc of 15° or more may be interpreted as meaningful functional gains following elective TEA. Across both single- and multi-item scales, we propose the use of the half SD-derived MCIDs, as we believe that they are more likely to be closer estimates of the true MCID of the instruments.^29^ From a research perspective, TEA-specific MCIDs can inform outcome reporting, facilitate comparison between studies, and support the design of adequately powered clinical trials. The lack of agreed MCID thresholds has historically limited the interpretability of statistically significant findings in elbow arthroplasty research. By providing data-driven estimates derived from contemporary elective TEA cohorts, this study contributes to standardizing outcome interpretation and improving the quality of evidence synthesis in this field.

A recent systematic review by Buchanan et al (2025)^4^ agrees with our findings, having shown that MEPS is by far the most used outcome measure in elbow arthroplasty. The authors call for more homogeneous reporting of outcome measures in future, including the use of the MEPS. We think the MEPS, despite elbow specific, is not an ideal instrument, as it is not exclusively patient-reported, which makes its widespread use more challenging, and the physician component may introduce examiner bias, especially in its “stability” category. Additionally, as we showed, its internal consistency is low, suggesting that its items are not closely related to each other about what they assess and this might influence its applicability and interpretation. We believe that a new, TEA-specific outcome measure should be devised, which should be exclusively patient-reported to optimize widespread use, capture the direct patient experience without potential for physician-driven bias, and use items which are relevant to the modern society and take into account population-specific considerations.

Important limitations warrant consideration. First, distribution-based MCIDs do not directly capture patient perception of meaningful change and should ideally be interpreted alongside anchor-based estimates when such data exist. Second, this study focused exclusively on elective TEA and does not address MCID thresholds for trauma-related or revision elbow arthroplasty, where baseline disability and patient expectations differ substantially. Separate condition-specific MCID work is likely required for these populations.

## Conclusions

This study provides TEA-specific, distribution-based MCID estimates for commonly used pain, functional, and motion outcome measures following primary elective TEA. The proposed thresholds are broadly consistent with existing elbow literature while offering improved relevance to contemporary elective TEA populations. In the absence of robust anchor-based data, these MCIDs offer a pragmatic framework for interpreting clinical outcomes, guiding research design, and enhancing meaningful evaluation of patient benefit after elbow arthroplasty. Future prospective studies incorporating patient-anchored measures are needed to further refine and validate these thresholds and a hybrid approach considering both anchor- and distribution-based methods should be used to define definitive MCIDs for primary elective TEA.

## Disclaimers:

Funding: Supported by MRC Grant MR/X036766/1.

Conflicts of interest: The authors, their immediate families, and any research foundations with which they are affiliated have not received any financial payments or other benefits from any commercial entity related to the subject of this article.
